# Trends in prevalence and determinants of severe and moderate anaemia among women of reproductive age during the last 15 years in India

**DOI:** 10.1371/journal.pone.0286464

**Published:** 2023-06-01

**Authors:** Marimuthu Sappani, Thenmozhi Mani, Edwin Sam Asirvatham, Melvin Joy, Malavika Babu, Lakshmanan Jeyaseelan

**Affiliations:** 1 Department of Biostatistics, Christian Medical College, Vellore, Tamil Nadu, India; 2 Health Systems Research India Initiative (HSRII), Trivandrum, Kerala, India; 3 Faculty of Medical Sciences, Translational and Clinical Research Institute, Newcastle University, Newcastle upon Tyne, United Kingdom; 4 Centre for Trials Research, College of Biomedical and Life Sciences, Cardiff University, Cardiff, United Kingdom; 5 Mohammed Bin Rashid University of Medicine and Health Sciences, Dubai Healthcare City, Dubai, UAE; University of Botswana, BOTSWANA

## Abstract

**Background:**

Anaemia is a serious global public health problem that disproportionally affects children, adolescent girls, and women of reproductive age, especially pregnant women. Women of reproductive age are more vulnerable to anaemia, particularly severe and moderate anaemia leads to adverse outcomes among pregnant women. Despite continuous Government efforts, anaemia burden still poses a serious challenge in India. The objective of this study is to assess the trends in prevalence and determinants of severe and moderate anaemia among women of reproductive age between 15 and 49 years.

**Method:**

We used three rounds of the large-scale National Family Health Survey (NFHS) India, conducted on a representative sample of households using a cross-sectional design across the country in 2005–06, 2015–16 and 2019–2021. We included all the women aged 15 to 49 years in our analysis. We used the same haemoglobin (Hb) cut-off values for all the three rounds of surveys to ensure comparability. Generalized linear regression analyses with log link were done. Survey weights were incorporated in the analysis.

**Results:**

The prevalence of severe or moderate Anaemia (SMA) in non-pregnant women was 14.20%, 12.43% and 13.98%; it was 31.11%, 25.98% and 26.66% for pregnant women in 2006, 2016 and 2021 respectively. The decline in SMA prevalence was 1.54% in non-pregnant women, whereas it was 14.30% in pregnant women in 15 years. Women who were poor, and without any formal education had a higher risk for severe and moderate Anaemia.

**Conclusion:**

Despite the intensive anaemia control program in India, SMA has not declined appreciably in non-pregnant women during the last two decades. Despite the decline, the prevalence of SMA was about 26% in pregnant women which calls for a comprehensive review of the existing anaemia control programmes and there must be targeted programmes for the most vulnerable and high-risk women such as rural, poor and illiterate women of reproductive age to reduce the burden of anaemia among them.

## Introduction

Anaemia is one of the highly prevalent health conditions and a major risk factor contributing significantly to the global burden of disease [1]. According to the World Health Organization (WHO), Anaemia is defined as having haemoglobin (Hb) levels lower than 11.0, 12.0, and 13.0 g/dL in pregnant women, non-pregnant women and men, respectively. It disproportionally affects children, adolescent girls, and women of reproductive age, especially pregnant women [2]. Due to the persistent reduction in oxygen-carrying capacity, anaemia can significantly reduce the cognitive, physical and work capacities and is associated with reduced economic productivity, increased susceptibility to infections due to its effect on immunity, increased morbidity and mortality [3–5]. Among pregnant women, iron-deficient anaemia can lead to adverse pregnancy outcomes, including stillbirth, preterm delivery, low birth weight, and infant mortality [6–8]. Moreover, anaemia can be a risk or a prognostic factor for other diseases, such as tuberculosis and heart failure [9,10].

Globally, the anaemia prevalence in women of reproductive age was 29.9%; equivalent to over half a billion women aged 15–49 years in 2019. The prevalence was relatively higher in pregnant women with 36.5% compared to non-pregnant women (29.6%) [11]. The prevalence of anaemia among women of reproductive age in the South Asia region was 41%, it was 48% in pregnant women and 49% in non-pregnant women in 2019 [12]. According to National Family Health Survey (NFHS)–IV (2015–16), the prevalence of anemia among women aged 15 to 49 years was 53.1% it was the 5^th^ highest among globally [13].

There have been consistent global efforts to address the burden of anaemia. For instance, the 65^th^ World Health Assembly (WHA) in 2012 approved global targets for maternal, infant, and young child nutrition, with a commitment to halve anaemia prevalence in women of reproductive age (15–49 years) by 2025. Following this, WHO and UNICEF proposed extending this target to 2030 to align with the UN Sustainable Development Goals (SDGs) 2- End hunger, achieve food security and improved nutrition and promote sustainable agriculture. The Government of India has also been taking several efforts to address the burden of anaemia among women especially anaemia among pregnant women. The Anaemia Mukt Bharat (AMB) which was launched in 2018 as part of the Strengthened Nationwide Iron Plus Initiative Project aims to lower the prevalence of anaemia by 1 to 3 percentage points each year, targeting children and women of reproductive age group [14]. Despite the significant efforts, 2/3^rd^ of all women of reproductive age in India are still having any form of anaemia (mild, moderate, and severe). Though, all types of anaemia must be given due importance, moderate and severe anaemia in non-pregnant women are to be treated with utmost care as significant health consequences are predominantly associated with moderate to severe anaemia [15]. In many cases, mild and asymptomatic anaemia require no management [16]. A recent study indicated that pregnant women with moderate and severe anaemia had higher risk for some adverse outcomes, including maternal shock, admission to the ICU, mortality, fetal growth restriction and stillbirth and increased risks were found among those with moderate or severe anaemia [17,18]. Severe anaemia is strongly correlated with maternal morbidity and mortality [19,20]. A study from central India highlighted similar risks from mild anaemia as well [21].

Besides, studies have widely reported the multiple risk factors associated with anaemia. For instance, rural residence; low socio-economic status such as eating <1 serving of meat/ week, farming and more number of children (>3 children); women with lower income level or wealth; lower education level; underweight women; women without toilet facilities or improved water facilities and women with more than one children had significantly higher risk for anaemia [22–27]. Though the exact link of BMI with anemia is controversial issue, several studies have highlighted that woman with higher BMI had greater likelihood of being anemic [28–30]. In addition, several clinical conditions, acute and chronic infections and diseases like Cancer, Chronic Kidney Disease, Malaria etc. reported to be associated with higher likelihood of anaemia [31–33].

Considering the high prevalence of anaemia among women in India, a focus on moderate to severe anaemia will be more appropriate to reduce the functional consequences and improve the overall health status of the women of reporductive health [15]. Therefore, our objective was to study the trends in prevalence and determinants of severe and moderate anaemia among women of reproductive age (15–49 years) using the three rounds of National Family Health Survey (NFHS-3, 4, and 5) which provides nationally representative cross-sectional data.

## Methods

We used three rounds of the large scale NFHS, conducted on representative sample of households across the country in 2005–06 (NFHS-3), 2015–16 (NFHS-4) and 2019–2021 (NFHS-5). The data was abstracted from https://dhsprogram.com/data/dataset_admin. The cross-sectional surveys collected detailed information on population, health and nutrition.

### Independent variables

The demographic, socioeconomic, cultural and behavioural covariates included in the analysis were age, place of residence, education, wealth, occupation, obesity, zone and parity. Age was categorised into four groups such as 15–19, 20–29, 30–39, and 40–49 years. Parity, defined as the number of children ever born, was categorised as 0, 1, 2, 3or more. Obesity was categorized as binary variable with BMI≥30.0 kg/m^2^. For wealth index, poorest and poor were combined as a category and rich and richest were combined as another category, but middle remains same. Education was categorized as no education, primary, secondary and higher education. Occupation of the respondent was classified as employed and unemployed. The states were grouped as north, east, west, south and north east [34].

### Dependent variables

The outcome variable haemoglobin adjusted for altitude and smoking was measured in g/dl and categorized as mild, moderate and severe anaemia based on predefined cut-off values as recommended by WHO. The cut-off values of mild, moderate, and severe anaemia for pregnant women were 10.00–10.90 g/dl, 7.00–9.90 g/dl, and <7.00 g/dl respectively in all the three rounds of NFHS. Among non-pregnant women, the cut-off values of mild, moderate and severe anaemia were 10.0–11.9 g/dl, 7.0–9.9 g/dl and < 7.0 g/dl in NFHS-3 and 4. In NFHS-5, the cut-off values for non-pregnant women were revised as 11.00–11.90 g/dl, 8.00–10.90 g/dl and <8.00 g/dl for mild, moderate and severe anaemia respectively [35]. As the cut-off levels have been revised in NFHS-5, we analysed the data using the same cut-off levels used in the previous rounds and presented the results for better comparison. As the severe and moderate anaemia require programmatic importance, they were combined for adjusted analysis.

Total number of women aged between 15 and 49 was 124,385, 699,686, and 724,115 in NFHS 3, 4, and 5 surveys respectively. In NFHS 3, Hb was measured among 112,714 (91%) women, it was not conducted in the state of Nagaland (3896, 3%) and in other states Hb value was not available for 7,775 (6%) women. In NFHS 4 and 5 surveys, 684,911 (98%) and 690,153 (95%) women were tested for Hb respectively. However, the Hb value was not available for 14,775 (2%) and 33962 (5%) women from NFHS 4 and 5 surveys due to various reasons.

**Statistical methods.** The variables were presented as frequency and percent for pregnant and non-pregnant women separately. Generalized linear model was used with log link as the prevalence was over 10%. The survey weights were incorporated in the analyses, which are provided in the NFHS data. The dependent variable anaemia was categorised as bivariate (Moderate and Severe vs. Mild and Normal). The model was repeated with the same covariate separately for pregnant and non-pregnant women. Negelkerke R^2^ and Hosmer and Lemeshow Goodness of Fit test was used to assess the model fit. Data was analysed using STATA software version 16.0. The survey (svy) command was used to weight the data in the regression analysis. The effect size is presented as risk ratio (RR) and 95% confidence intervals.

#### Ethical considerations

Informed consent was obtained from participants at the time of interview, and further consent was obtained prior to blood testing as per the NFHS protocol. All survey participants were provided an informational leaflet at the time of anaemia testing; women diagnosed with severe anaemia were asked if they could be referred to local health services. The analysis was approved by Institutional Review Board of Christian Medical College, Vellore, India.

## Results

Table 1 presents the prevalence of different levels of anaemia by the year of NFHS and pregnancy status. Considering similar Hb cut-off level for all three rounds, the prevalence of severe anaemia (SA) was about 1.56%, 1.01% and 1.17% in 2006, 2016 and 2021 respectively. However, in non-pregnant women the reported prevalence of SA was 2.64% in 2021, which is a significant increase from the previous rounds due to the revised Hb cut-off level. The prevalence of moderate anaemia (MoA) was 13.43%, 12.04% and 13.31% in 2006, 2016 and 2021 respectively. Both, SA and MoA prevalence declined in 2016 and increased in 2021. Mild anaemia (MA) indicated a marginal increase from 2006 to 2016 and 2021.

**Table 1:** Anaemia among women of reproductive age group by pregnancy status in NFHS 3, 4, and 5.

Variables	Anaemia				
	Severe	Moderate	Mild	Not anaemic	Total
**Over all**					
NFHS 3 (2006)	1763 (1.56)	15138 (13.43)	41749 (37.04)	54064 (47.97)	112714
NFHS 4 (2016)	6950 (1.01)	82490 (12.04)	263132 (38.42)	332339 (48.52)	684911
NFHS 5 (2021)	8078 (1.17)	91856 (13.31)	287865 (41.71)	302354 (43.81)	690153
NFHS 5- New definition	18221 (2.64)	195685 (28.35)	173893 (25.20)	302354 (43.81)	690153
**Non Pregnant**					
NFHS 3 (2006)	1650 (1.54)	13597 (12.66)	40402 (37.62)	51748 (48.18)	107397
NFHS 4 (2016)	6489 (0.99)	74679 (11.44)	255598 (39.14)	316297 (48.43)	653063
NFHS 5 (2021)	7657 (1.16)	84995 (12.82)	281356 (42.45)	288828 (43.57)	662836
NFHS 5 -New definition	17800 (2.69)	188824 (28.49)	167384 (25.25)	288828 (43.57)	662836
**Pregnant**					
NFHS 3 (2006)	113 (2.13)	1541 (28.98)	1347 (25.33)	2316 (43.56)	5317
NFHS 4 (2016)	461 (1.45)	7811 (24.53)	7534 (23.66)	16042 (50.37)	31848
NFHS 5 (2021)	421 (1.54)	6861 (25.12)	6509 (23.83)	13526 (49.51)	27317

Note: The definition for Anaemia has been changed for non-pregnant women only.

### Prevalence of anaemia among non-pregnant women

The prevalence of SA was 1.54%. 0.99% and 1.16% in 2006, 2016 and 2021 respectively. According to the new definition there was about 2 times increase in the prevalence of SA in 15 years. The prevalence of moderate and mild anaemia also showed the similar trends. The prevalence of MoA was about 12.66%, 11.44% and 12.82% in 2006, 2016 and 2021 surveys respectively.

### Prevalence of anaemic among pregnant women

The prevalence of SA was 2.13%, 1.45% and 1.54% in 2006, 2016 and 2021 surveys respectively. The prevalence of MoA was about 28.98%, 24.53% and 25.12% in 2006, 2016 and 2021 surveys respectively.

### Prevalence of severe & moderate anaemia in non-pregnant and pregnant women by socio-economic, demographic variables

The prevalence of severe and moderate anaemia (SMA) is presented by covariates such as age, residence, education, wealth, obesity, zone, occupation and Parity. In Table 2, among non-pregnant women, the prevalence of SMA was nearly similar for all age categories in all three rounds of NFHS including the adolescent women aged 15 to19 years. Women without formal education, rural, economically poor, and women without any children reported higher prevalence of SMA compared to women with some formal education, urban and economically wealthy (middle or rich) women, and having at least one child in all the three rounds. Interestingly, employed women reported a higher prevalence of SMA compared to unemployed women. On the other hand, obese women indicated relatively less prevalence of SMA compared non-obese women. The North East region reported lower prevalence in 2016 and 2021as compared to other regions.

**Table 2:** Descriptive statistics for Anaemia among non-pregnant women.

Variable	NFHS 3 (2006)			NFHS 4 (2016)			NFHS 5 (2021)		
	Severe & Moderate	Mild	Total	Severe & Moderate	Mild	Total	Severe & Moderate	Mild	Total
	n (%)	n (%)	n	n (%)	n (%)	n	n (%)	n (%)	N
**Age**									
15–19	2962 (14.32)	7950 (38.44)	20681	14250 (12.06)	47586 (40.29)	118123	16162 (14.32)	50060 (44.36)	112837
20–29	5042 (14.20)	13545 (38.15)	35509	25993 (12.39)	83681 (39.88)	209813	28289 (13.66)	89426 (43.17)	207149
30–39	4200 (13.85)	11180 (36.87)	30320	22126 (12.35)	68519 (39.26)	179094	25836 (13.89)	77476 (41.65)	185996
40–49	3043 (14.57)	7727 (36.99)	20887	18799 (12.87)	55812 (38.22)	146033	22365 (14.26)	64394 (41.05)	156854
**Type of Place of residence**									
Urban	6113 (12.77)	17227 (36.00)	47851	21821 (11.47)	70785 (37.20)	190289	19979 (12.34)	64960 (40.13)	161868
Rural	9134 (15.34)	23175 (38.92)	59546	59347 (12.83)	184813 (39.94)	462774	72673 (14.51)	216396 (43.2)	500968
**Highest educational level**									
No education	5952 (17.18)	13941 (40.24)	34645	26661 (14.39)	76133 (41.11)	185194	23882 (15.39)	68152 (43.93)	155149
Primary	2450 (15.76)	5989 (38.51)	15550	10726 (12.99)	32473 (39.33)	82573	11576 (14.74)	33623 (42.83)	78509
Secondary	5869 (12.63)	16908 (36.40)	46446	36724 (11.78)	119937 (38.44)	311982	46960 (13.89)	143520 (42.43)	338247
Higher	975 (9.07)	3560 (33.13)	10745	7057 (9.63)	27055 (36.9)	73314	10234 (11.25)	36061 (39.66)	90931
**Wealth**									
Poor	4993 (18.10)	11764 (42.64)	27589	35525 (13.49)	110023 (41.79)	263260	42437 (14.88)	128411 (45.01)	285295
Middle	3163 (15.37)	7822 (38.01)	20581	17411 (12.61)	52242 (37.84)	138063	19950 (14.26)	57921 (41.41)	139880
Rich	7091 (11.98)	20816 (35.15)	59227	28232 (11.21)	93333 (37.08)	251740	30265 (12.74)	95024 (39.98)	237661
**Obesity**									
Non Obese	14892 (14.40)	39064 (37.78)	103387	78304 (12.56)	245336 (39.33)	623766	88262 (14.10)	266929 (42.64)	625977
Obese	330 (8.50)	1288 (33.20)	3880	2729 (9.56)	9992 (34.98)	28567	4235 (11.76)	14064 (39.05)	36013
**Zone**									
North	4083 (13.30)	10749 (35.02)	30694	26300 (13.01)	77384 (38.28)	202137	30952 (14.22)	88022 (40.44)	217642
East	2498 (15.35)	7750 (47.63)	16272	15562 (13.24)	55572 (47.28)	117540	16376 (15.08)	55721 (51.3)	108609
North East	2173 (13.34)	6226 (38.22)	16288	7317 (7.96)	28336 (30.84)	91873	10203 (10.65)	38263 (39.92)	95841
West	3270 (13.73)	8579 (36.01)	23821	16382 (12.13)	53775 (39.8)	135120	19764 (14.47)	60652 (44.42)	136539
South	3223 (15.86)	7098 (34.93)	20322	11875 (13.77)	31645 (36.7)	86235	15357 (14.74)	38698 (37.14)	104205
**Occupation**									
Unemployed	8596 (13.61)	23631 (37.43)	63136	10487 (13.37)	30391 (38.75)	78431	9120 (13.61)	27766 (41.43)	67011
Employed	6638 (15.03)	16733 (37.88)	44170	4980 (14.68)	13161 (38.79)	33929	4804 (14.71)	13290 (40.72)	32639
**Parity**									
No children	241 (17.81)	486 (35.92)	1353	4334 (13.19)	12549 (38.18)	32866	4702 (14.93)	13239 (42.03)	31498
Single Child	1347 (12.35)	4032 (36.96)	10910	4562 (11.31)	15597 (38.66)	40345	5657 (13.53)	17698 (42.33)	41805
Two Children	8943 (14.02)	23909 (37.49)	63766	48259 (12.46)	153599 (39.64)	387507	59779 (14.23)	180243 (42.9)	420120
3+ Children	4220 (14.89)	10908 (38.49)	28342	23367 (12.50)	71829 (38.43)	186917	21390 (13.19)	67146 (41.42)	162120

The prevalence of SMA among pregnant women by covariates is presented in Table 3. Among pregnant women who were aged 40–49 years indicated relatively higher SMA prevalence compared to other age groups especially in 2006, and 2016. Similar to non-pregnant women, rural pregnant women, women without formal education, economically poor had consistently higher prevalence of SMA compared to urban, women with some formal education, and wealthy. Besides, obese women indicated relatively lower prevalence of SMA compared to non-obese women.

**Table 3:** Descriptive statistics for Anaemia among pregnant women.

Variables	NFHS 3 (2006)			NFHS 4 (2016)			NFHS 5 (2021)		
	Severe & Moderate	Mild	Total	Severe & Moderate	Mild	Total	Severe & Moderate	Mild	Total
	n (%)	n (%)	n	n (%)	n (%)	n	n(%)	n(%)	n
**Age**									
15–19	283 (29.45)	262 (27.26)	961	932 (25.84)	916 (25.40)	3607	828 (28.76)	755 (26.22)	2879
20–29	1109 (30.93)	893 (24.90)	3586	5980 (25.83)	5509 (23.80)	23149	5255 (26.89)	4687 (23.98)	19546
30–39	243 (33.75)	182 (25.28)	720	1256 (26.32)	1051 (22.02)	4772	1129 (24.30)	1015 (21.84)	4647
40–49	19 (38.00)	10 (20.00)	50	104 (32.50)	58 (18.13)	320	70 (28.57)	52 (21.22)	245
**Type of place of residence**									
Urban	545 (27.78)	504 (25.69)	1962	1722 (22.73)	1716 (22.65)	7576	1158 (21.91)	1153 (21.82)	5285
Rural	1109 (33.06)	843 (25.13)	3355	6550 (26.99)	5818 (23.97)	24272	6124 (27.80)	5356 (24.31)	22032
**Highest educational level**									
No education	760 (38.58)	496 (25.18)	1970	2647 (32.95)	1996 (24.85)	8033	1529 (34.03)	1127 (25.08)	4493
Primary	246 (31.74)	188 (24.26)	775	1212 (28.67)	972 (23)	4227	903 (30.27)	722 (24.2)	2983
Secondary	571 (26.64)	557 (25.99)	2143	3732 (23.64)	3781 (23.95)	15790	4007 (25.94)	3706 (23.99)	15445
Higher	77 (17.95)	106 (24.71)	429	681 (17.93)	785 (20.67)	3798	843 (19.18)	954 (21.7)	4396
**Wealth**									
Poor	731 (39.07)	482 (25.76)	1871	4541 (30.04)	3724 (24.64)	15114	3975 (30.31)	3314 (25.27)	13114
Middle	345 (31.97)	265 (24.56)	1079	1590 (24.50)	1575 (24.27)	6490	1404 (25.33)	1286 (23.2)	5542
Rich	578 (24.42)	600 (25.35)	2367	2141 (20.90)	2235 (21.82)	10244	1903 (21.97)	1909 (22.04)	8661
**Obesity**									
Non Obese	1638 (31.34)	1318 (25.22)	5226	8076 (26.08)	7352 (23.75)	30962	7019 (26.80)	6247 (23.85)	26194
Obese	14 (16.09)	28 (32.18)	87	188 (21.68)	176 (20.3)	867	257 (23.53)	254 (23.26)	1092
**Zone**									
North	508 (30.46)	386 (23.14)	1668	2879 (27.47)	2308 (22.02)	10481	2302 (25.24)	2025 (22.21)	9119
East	313 (33.73)	280 (30.17)	928	1910 (29.70)	1776 (27.62)	6431	1734 (33.43)	1483 (28.59)	5187
North East	260 (30.99)	197 (23.48)	839	764 (17.06)	903 (20.16)	4479	937 (20.40)	953 (20.74)	4594
West	349 (31.30)	282 (25.29)	1115	1691 (27.02)	1570 (25.08)	6259	1517 (29.62)	1257 (24.54)	5122
South	224 (29.20)	202 (26.34)	767	652 (21.72)	680 (22.65)	3002	792 (24.04)	791 (24.01)	3295
**Occupation**									
Unemployed	1100 (30.27)	918 (25.26)	3634	1157 (27.19)	1020 (23.97)	4255	845 (27.25)	706 (22.77)	3101
Employed	553 (32.92)	428 (25.48)	1680	319 (29.70)	255 (23.74)	1074	222 (26.52)	186 (22.22)	837
**Parity**									
No children	10 (37.04)	5 (18.52)	27	177 (26.18)	153 (22.63)	676	146 (26.31)	117 (21.08)	555
Single Child	84 (24.00)	92 (26.29)	350	271 (21.93)	259 (20.95)	1236	261 (23.92)	230 (21.08)	1091
Two Children	938 (30.21)	792 (25.51)	3105	4906 (25.29)	4606 (23.74)	19402	4716 (26.90)	4284 (24.44)	17530
3+ Children	578 (33.39)	440 (25.42)	1731	2869 (27.62)	2488 (23.95)	10387	2060 (26.21)	1823 (23.19)	7860

### Factors associated with the severe and moderate anaemia among women of reproductive age

The results of multivariable analysis are presented in Table 4. In non-pregnant women, the risk of SMA in 2016 declined by 18% from 2006 and declined in 2021 by 7%. Wealth status, education, obesity and region were significantly associated with the prevalence of SMA. Poor and middle-class women had 16% (RR: 1.16; CI: 1.14–1.18) and 12% (RR: 1.12; CI: 1.10–1.14) higher risk of having SMA compared to rich women. Similarly, those who had no education (RR: 1.35; CI: 1.31–1.39) or primary (RR: 1.31; CI: 1.27–1.35) or secondary education (RR: 1.21; CI: 1.18–1.25) had higher risk for SMA compared to those women who had higher education. The non-obese women had 1.21 times (RR: 1.21; CI: 1.17–1.25) more risk of having SMA as compared to obese women. Compared to north region, North East women had less risk (RR: 0.92; CI: 0.89–0.95) of having SMA and women from East (RR: 1.07; CI: 1.05–1.10), West (RR: 1.06; CI: 1.03–1.08) and Southern region (RR: 1.21; CI: 1.19–1.24) had higher risk of having SMA.

**Table 4:** Multivariable analysis results (GLM with log link).

Variables	Non Pregnant Women		Pregnant Women	
	Risk Ratio (95% CI)	P value	Risk Ratio (95% CI)	P value
**NFHS Survey**				
**NFHS 3**	Ref		Ref	
**NFHS 4**	0.82 (0.80, 0.84)	<0.001	0.83 (0.79, 0.88)	<0.001
**NFHS 5**	0.93 (0.90, 0.96)	<0.001	0.94 (0.88, 0.99)	0.029
**Age**				
**15–19**	0.99 (0.97, 1.01)	0.515	0.88 (0.74, 1.05)	0.147
**20–29**	0.99 (0.97, 1.002)	0.095	0.93 (0.78, 1.10)	0.393
**30–39**	0.97 (0.96, 0.99)	0.001	0.94 (0.79, 1.11)	0.446
**40–49**	Ref		Ref	
**Residence**				
**Rural**	1.01 (0.99, 1.03)	0.219	1.02 (0.96, 1.08)	0.531
**Urban**	Ref		Ref	
**Wealth**				
**Poor**	1.16 (1.14, 1.18)	<0.001	1.28 (1.21, 1.35)	<0.001
**Middle**	1.12 (1.10, 1.14)	<0.001	1.17 (1.10, 1.24)	<0.001
**Rich**	Ref		Ref	
**Education**				
**No education**	1.35 (1.31, 1.39)	<0.001	1.61 (1.48, 1.75)	<0.001
**Primary**	1.31 (1.27, 1.35)	<0.001	1.50 (1.37, 1.64)	<0.001
**Secondary**	1.21 (1.18, 1.25)	<0.001	1.33 (1.24, 1.44)	<0.001
**Higher**	Ref		Ref	
**Obesity**				
**Non Obese**	1.21 (1.17, 1.25)	<0.001	1.01 (0.89, 1.13)	0.912
**Obese**	Ref		Ref	
**Zone**				
**North**	Ref		Ref	
**East**	1.07 (1.05, 1.10)	<0.001	1.08 (1.03, 1.13)	0.001
**North East**	0.92 (0.89, 0.95)	<0.001	0.93 (0.86, 0.99)	0.028
**West**	1.06 (1.03, 1.08)	<0.001	1.10 (1.05, 1.16)	0.000
**South**	1.21 (1.19, 1.24)	<0.001	0.98 (0.92, 1.04)	0.451

Among pregnant women, the risk of SMA declined by 17% and 6% in 2016 and 2021 surveys respectively (p < .001). Like non-pregnant women, wealth status, education, and region except obesity were significantly associated with the prevalence of SMA. Poor and middle-class women had 28% (RR: 1.28; CI: 1.21–1.35) and 17% (RR: 1.17; CI: 1.10–1.24) higher risk of having SMA compared to rich women. Similarly, those who had no education (RR: 1.61; CI: 1.48–1.75) or primary (RR: 1.50; CI: 1.37–1.64) or secondary education (RR: 1.33; CI: 1.24–1.44) had higher risk for SMA compared to those women with higher education. Compared to north region, north east women had less risk (RR: 0.93; CI: 0.86–0.99) of having SMA and women from east (RR: 1.08; CI: 1.03–1.13), and west (RR: 1.10; CI: 1.05–1.16) had risk of having SMA.

## Discussion

Anaemia is largely preventable and easily treatable if the determinants at the local and national level are identified, appropriate strategies are devised and implemented to combat anaemia recognising its multi factorial etiology [24]. The findings of the last three rounds of NFHS in India indicated that the prevalence of any anaemia which includes severe, moderate and mild anaemia among women of reproductive age increased significantly from 52% to 56% in 15 years, though there was a slight decline in 2016. According to WHO, the current situation falls under the severe category of public health significance (prevalence >40%) [36]. It is evident that there has been little or no progress in reducing anaemia among women over the past two decades. Especially, the prevalence of severe and moderate anaemia (SMA) remains almost similar during the last 15 years among non-pregnant women; however it declined significantly among pregnant women (14% decline in 15 years). At the same time, prevalence of SMA was still considerably high at 27% among pregnant women as compared to non-pregnant women which has enormous programmatic importance and implications in the country. These trends and patterns were almost similar across all socioeconomic groups. The increase in the prevalence of any anaemia and the consistent high prevalence of SMA over the last 15 years, despite the comprehensive anaemia policy framework, intensive programmatic efforts of the state and central governments, significant economic development and increase in the investment in health is a real concern. This could be due to the poor implementation and targeting that lead to poor coverage of potential beneficiaries of the National Anaemia Control Programme (NACP) and National Iron plus Initiative (NIPI) guidelines [37,38].

Importantly, almost half of the pregnant women in India had any anaemia and over a quarter of them (27%) had SMA as per the NFHS 2021, which is the highest prevalence of anaemia in pregnancy and the largest number of anaemia pregnant women worldwide [39]. Despite the current higher prevalence of SMA, the significant decline of it over the last 15 years among pregnant women could be due the focused anaemia control programmes among pregnant women in India. Moreover, there have been significant improvements in the nutrition and health of women, increasing utilisation of antenatal care and iron and folic acid supplementation, increasing use of contraception, as well as increased age at marriage and decreased total fertility rate over the years [37]. In specific, the previous rounds of NFHS have indicated improvement in coverage of iron-folic acid supplementation and ANC which could have had an effect in the reduction of SMA among pregnant women [40].

The study also revealed that anaemia especially SMA disproportionally affects the socio-economically vulnerable women of reproductive age group in the country. For instance, the higher prevalence of SMA among illiterate, rural, and economically poor among both pregnant and non-pregnant women indicates the persistence inequalities in the health status of women which could be due to the inequalities in coverage and access to anaemia control interventions among these groups. These findings corroborate with several other studies carried out in India and other less-developed and developing countries that indicate that anaemia disproportionately affect the rural, poor, less educated and other socially vulnerable population [41–44]. The NFHS indicated a higher prevalence of SMA among women without any children, however, it indicated an increasing trend with number of children. This pattern in agreement with several other studies that highlighted high parity as a risk factor for developing severity of iron deficiency anaemia in pregnancy [45–47].

Interestingly, employed pregnant and non-pregnant women reported a higher prevalence of SMA compared to unemployed women in all the three rounds of NFHS, except pregnant women in 2021. Though employment and socio-economic status of the women are correlated, the reasons for the higher rates of SMA among unemployed women while the prevalence of SMA was higher among illiterate and poor women are not clearly known and need to be studied further. On the other hand, obese women reported low prevalence of SMA compared to non-obese women which is in corroboration with numerous studies across the word [29,48]. However, several other studies have indicated either no difference or higher prevalence of anaemia among obese women [48]. A cross-sectional study conducted in Israel in 2003 showed a higher prevalence of iron deficiency in overweight and obese children and adolescents [49]. Few other studies reported an increase in the prevalence of iron deficiency in obese adults with significantly lower serum iron level and higher soluble transferrin receptor level than non-obese adults [50,51].

### Limitations

Several studies have reported significant association between anaemia and many diseases, clinical conditions, and infections. However, we could not include them in our analysis as the focus of the paper was limited to the burden of severe and moderate anaemia and their socio-economic and demographic correlates. Being a cross sectional study, the cause-and-effect relationship could not be established. For instance, there is a strong association between socio-economic situation and anaemia, which could be bidirectional. Though systems are calibrated against standard tool, the upgraded model of analyser to measure Hb used in subsequent surveys could have affected the Hb measurements during the different surveys [27].

## Conclusion

The analysis of three rounds of NFHS indicated that there has been little or no progress in the reduction of anaemia, despite the intensive programmatic efforts in the country. Especially, the consistent high prevalence of severe or moderate anaemia among women of reproductive age groups over the past two decades is a serious concern which would lead to several complications and consequences. The reduction of severe and moderate anaemia among pregnant women could be due to the programmatic efforts. However, SMA prevalence is unacceptably high among pregnant women compared to non-pregnant women which call for urgent targeted programmes among pregnant women to accelerate decline in anaemia in pregnancy. Universal testing, measures for reducing anaemia and early initiation of treatment in pregnant women are critical to combat the issue among pregnant women. Similarly, targeted efforts are required to address the consistent problem of SMA among non-pregnant women of reproductive age group. The analysis clearly indicated that women’s education and socio-economic improvement of women are the most important determinants of anaemia control among women of reproductive age group which must be addressed through appropriate structural interventions to improve and ensure universal coverage of anaemia control programmes in the country. The study also indicated regional variability in terms of severe and moderate anaemia which need to be studied further developing appropriate regional specific strategies. Considering the high proportion of mild anaemia, policies and programs aiming at reducing the severe and moderate anaemia will be more effective and relevant to improve the overall health and productivity of women in India.

## Abbreviations

WHO: World Health Organization
Hb: Haemoglobin
NFHS: National Family Health Survey
RR: Risk ratio
SA: Severe Anaemia
MoA: Moderate Anaemia
MA: Mild Anaemia
SMA: Severe and Moderate anaemia
